# Correction Notice: Correlative Conventional and Super-resolution Photoactivated Localization Microscopy (PALM) Imaging to Characterize Chromatin Structure and Dynamics in Live Mammalian Cells

**DOI:** 10.21769/BioProtoc.4896

**Published:** 2023-11-05

**Authors:** Dushyant Mehra, Elias M. Pucher

**Affiliations:** 1 School of Physics and Astronomy, University of Minnesota, Twin Cities, MN, USA; 2 Department of Physiology and Biomedical Engineering, Mayo Clinic, Rochester, MN, USA

After publishing the protocol (https://bio-protocol.org/e4850), we noticed that the last name of the corresponding author was misspelled. **It should be Puchner**, not Pucher.

